# A review of deep learning approaches for drug synergy prediction in cancer

**DOI:** 10.1038/s44386-025-00034-1

**Published:** 2025-12-16

**Authors:** Lei Li, Hongyu Zhang, Chunhou Zheng, Yansen Su

**Affiliations:** 1 School of Artificial Intelligence, Anhui University, Hefei, China; 2 Institute of Artificial Intelligence, Hefei Comprehensive National Science Center, Hefei, China

**Keywords:** Drug discovery, Drug screening

## Abstract

Synergistic drug combinations enhance cancer treatment by improving efficacy and reducing toxicity. With advances in artificial intelligence and large-scale datasets, deep learning has become central to anti-cancer drug synergy prediction. This review summarizes classical and emerging deep learning models from single-task learning and multi-task learning perspectives, discusses data and technical challenges, and highlights future directions for advancing computational drug synergy prediction.

## Introduction

**Fig. 1:**
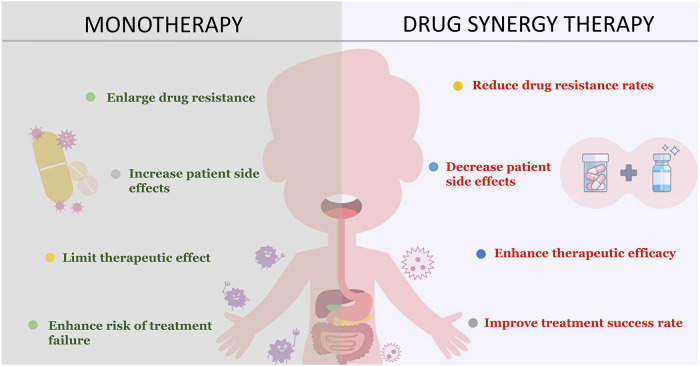
Comparison between monotherapy and drug synergy therapy. Drug synergy therapy has the advantages of reducing drug resistance rates, enhancing therapeutic efficacy, decreasing patient side effects, and improving treatment success rates compared to monotherapy.

Cancer stands as a highly complex disease with a staggering mortality rate worldwide, which poses a significant obstacle to the enhancement of life expectancy^1^. According to the statistical data of World Health Organization, approximately 9.7 million deaths were attributed to cancer in 2022^2^. Drug therapy, which has gained increasing attention in recent years, has become an important means of conquering cancer^3^. Traditional monotherapy for cancer treatment has limitations such as enlarged drug resistance, increased patient side effects, limited therapeutic effect, and enhanced risk of treatment failure^4^. Pharmacologically, drug combinations can lead to synergistic, additive, or antagonistic effects, depending on whether their combined impact exceeds, matches, or falls short of the individual effects of each drug^5^. Synergistic effects are particularly sought after in drug therapy as they reduce drug resistance rates, enhance therapeutic efficacy, decrease patient side effects, and improve treatment success rates. As shown in Fig. 1, the drug synergy therapy can effectively compensate for the shortcomings of monotherapy. Therefore, drug synergy therapy has become increasingly favored for its advantage to mitigate the side effects and resistance often encountered with the conventional ‘one drug-one target’ therapeutic strategy^6^. Concurrently administered drugs have demonstrated the ability to overcome biological compensation mechanisms and diminish unintended off-target effects. Over the past few years, a growing number of fixed-dose combination drugs have received Food and Drug Administration (FDA) approval for treating complex conditions, including cardiovascular diseases, type 2 diabetes, HIV, respiratory illnesses, neurodegenerative disorders, and cancer^7^. In the field of cancer therapy, the FDA granted approval for the first combination therapy in January 2014, targeting melanoma patients with BRAF V600E or V600K mutations^8^. Subsequently, more than 50 non-fixed dosage combination treatments have been sanctioned by the FDA for various cancer subtypes.

Numerous well-known in vitro computational approaches have been developed to detect synergistic effects of drug combinations. These approaches, such as Loewe additivity (Loewe)^9^, Bliss independence (Bliss)^10^, highest single agent (HSA)^11^ and zero interaction potency (ZIP)^12^, typically employ statistical methods based on drug dose data and phenotypic effects (e.g., cell death, viability, and growth rate) to calculate the synergy scores of drug combinations. However, these approaches cannot provide detailed information on how drugs interact at the molecular level, which limits a deep understanding of the mechanisms of drug synergy. Moreover, these approaches are often considered time-consuming because they typically identify only a limited range of synergistic drug effects through experimental trials.

To address the shortcomings of in vitro approaches, traditional machine learning-based models have been proposed to predict synergistic drug combinations. To enhance the efficiency of synergy prediction, Gayvert et al.^13^ simplified the efficacy of single drugs to the *GI*_50_ value, which is the concentration of the drug required to inhibit 50% of cell growth. They designed a random forest classifier to predict synergistic drug combinations based on the mean and difference of the *GI*_50_ values for single drugs. Subsequently, ComboFM^14^ applied a higher-order factorization machine to model the cell line-specific responses to a drug combination as an interaction between different tensor modes. To improve computational efficiency, ComboLTR^15^ can describe the responses of therapeutic drug combinations in various doses and cell line contexts. Notably, it combines drug chemical information and cell line multi-omics information with recommender system-style information that indexes the data tensor of response values for prediction. Compared to in vitro approaches to detect drug synergy, traditional machine learning-based prediction models offer the benefits of being less labor-intensive, time-efficient, and cost-effective. However, traditional machine learning-based prediction models still suffer from the lack of interpretability and insufficient prediction accuracy.

Deep learning-based prediction models have shown promise in inferring synergistic drug combinations. On the one hand, advancements in interpretability techniques for deep learning, such as attention mechanisms, feature visualization, and gradient analysis, have made the decision-making process of deep learning-based prediction models more transparent^16–18^. On the other hand, deep learning-based prediction models enhance prediction accuracy by employing efficient strategies that consolidate a wide array of biomedical data, including chemical structures of drugs, genomics of cell lines, drug-target interactions, cell line-target interactions, protein-protein interactions (PPIs), and other relevant information. By learning effective feature representations from heterogeneous biological sources, such models not only improve predictive power but also reduce the reliance on exhaustive experimental measurements. In practice, it is often infeasible to perform large numbers of costly and time-consuming assays to test every possible drug pair and cellular context^19^. Instead, deep learning models can infer potential interactions from existing heterogeneous data, thereby reducing the need for extensive information collection from multiple sources. This representation learning capability enables researchers to efficiently prioritize the most promising drug combinations for experimental validation, saving both time and resources while maintaining high accuracy^20^. Notably, these prediction models have primarily focused on drug synergy prediction in cancer, mainly because this disease has a well-established foundation of cell line screening data. Nevertheless, the underlying modeling principles are general and can also be applied to other diseases once sufficient synergy or omics data become available. In addition, the data used for drug synergy prediction can also be applied to other types of prediction tasks, such as drug-drug interaction (DDI) prediction and single-drug sensitivity prediction. These tasks can serve as auxiliary tasks in multi-task learning models to help learn more robust feature representations and enhance the performance of drug synergy prediction^21^. Existing deep learning-based prediction models can generally be grouped into single-task learning models and multi-task learning models. Single-task learning models focus exclusively on predicting drug synergy, utilizing features tailored to this end. In contrast, multi-task learning models identify and exploit features that are simultaneously important for drug synergy prediction and auxiliary tasks, thereby improving robustness and reducing the impact of noise from individual tasks.

**Fig. 2:**
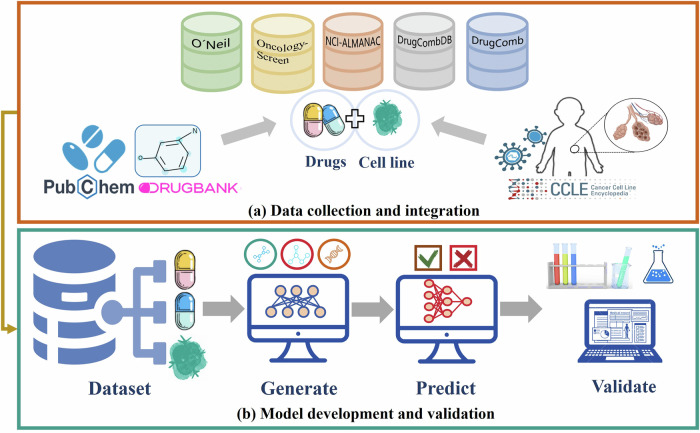
Deep learning model applications in the drug synergy prediction pipeline. The workflow consists of two major stages: **a** Data collection and integration. Samples are collected and integrated from public drug synergy databases, while the corresponding molecular and cellular attributes are retrieved from drug and cell line resources. **b** Model development and validation. The constructed dataset is used to generate feature representations, train deep learning models for drug synergy prediction, and validate the predicted results through wet-lab or computational methods.

In this review, we describe the classical and state-of-the-art deep learning-based drug synergy prediction models, with a particular focus on multi-task learning models, and discuss the limitations and challenges in this field. Figure 2 clearly illustrates the process of drug synergy prediction using deep learning-based models. The rest of this review is organized as follows. In Section “Drug synergy resources”, we introduce the existing drug synergy resources. In Section “Features of drugs and cell lines”, we describe the feature representations of drugs and cell lines. Section “Deep learning-based drug synergy prediction models” presents deep learning-based drug synergy prediction models, covering both single-task and multi-task learning approaches, along with a comparison of different model types. Finally, Section “Discussion and outlook” provides a discussion and outlook on current challenges and potential directions for future research.

## Drug synergy resources

Deep learning prediction models rely on a wide range of resources to capture complex relationships and patterns among drugs. In recent years, many research organizations dedicated to drug synergy research have developed evaluation metrics, databases, interactive software tools, and web portals that facilitate the discovery and analysis of synergistic drug combinations^19,20^.

### Evaluation metrics for drug synergy

Quantitative evaluation of drug synergy is fundamental for constructing reliable datasets and benchmarks in computational drug combination studies. Several reference models, including Loewe^9^, Bliss^10^, HSA^11^, and ZIP^12^, have been proposed to estimate the expected combined response assuming no interaction between the two drugs. In addition, various derived metrics, including ComboScore^22^ and Synergy (S) score^23^, have been proposed to quantify the deviation between the observed and expected effects based on these models.

The Loewe model assumes that a drug cannot interact with itself and that two drugs with identical mechanisms should exhibit an additive effect. It quantifies synergy by comparing the observed combination response to the expected response under the assumption of dose equivalence^9^. However, this method requires complete dose-response curves for both single agents and is less suitable when such data are unavailable.

The Bliss model assumes that two drugs act independently, and synergy is observed when the combined inhibition exceeds the expected independent effect^10^. Although conceptually simple, Bliss tends to overestimate synergy when two drugs act on the same target or pathway.

The HSA model^11^ defines synergy when the combination effect exceeds the maximum inhibition achieved by any of the individual drugs. It is easy to interpret but can underestimate synergy when both drugs individually exhibit weak efficacy.

The ZIP model integrates the concepts of Bliss and Loewe by comparing the entire dose-response surface of the observed combination to that expected under the assumption of no interaction^12^. It provides a robust and interpretable estimation of drug synergy and has been widely used in large-scale pharmacogenomic studies.

The ComboScore is a synergy evaluation metric derived from the difference between the observed drug combination response and the expected additive effect, often computed using models such as Bliss or Loewe. A higher ComboScore indicates a stronger synergistic effect, while a lower value reflects antagonism^22^. This metric has been widely adopted in large-scale pharmacogenomic studies due to its simplicity and interpretability, but it can be sensitive to the choice of underlying reference model.

The S score provides a simple metric of synergy by quantifying the percentage of inhibition that exceeds the expected additive effect when two drugs are combined at doses corresponding to their respective *IC*_50_ values^23^. Higher S scores indicate stronger synergistic inhibition, although this metric is limited to a single dose combination.

In summary, these synergy evaluation metrics offer complementary perspectives on drug-drug relationships. Loewe and Bliss focus on theoretical reference models, HSA emphasizes empirical comparison, and ZIP integrates both conceptual and experimental perspectives. The careful selection and consistent application of these metrics are essential for building reproducible and comparable benchmarks in drug synergy prediction research.

### Drug synergy datasets

Several commonly used drug synergy datasets, including O’Neil^24^, Oncology-Screen (On-Screen)^25^, NCI-ALMANAC^22^, DrugCombDB^26^, and DrugComb^27^, comprise thousands of drug combinations tested across multiple cancer cell lines and have been specifically curated to train deep learning-based prediction models. Detailed information on these datasets is provided in Table 1.

**Table 1:** Overview of benchmark drug synergy datasets, including the number of cell lines, drugs, samples, and the evaluation metrics used for synergy assessment

Source	No. of cell lines	No. of drugs	No. of samples	Evaluation metric
O’Neil	39	38	583	HSA, Bliss
On-Screen	29	21	4176	ZIP
NCI-ALMANAC	60	104	304,549	ComboScore
DrugCombDB	124	2887	448,555	HSA, Bliss, Loewe, ZIP
DrugComb	2320	8397	739,964	HSA, Bliss, Loewe, ZIP, S

Note: “No.” stands for “Number of”.

As the first dataset for training deep learning-based prediction models, the O’Neil dataset^24^ is derived from a large-scale oncology screen for 583 drug combination samples in 39 diverse cancer cell lines and 38 unique drugs. For combination screening, drugs are tested in a 4 by 4 matrix of drug concentrations representative of the cell-active concentrations of each drug. Synergy scores in the O’Neil dataset are reported using the HSA and Bliss metrics. However, computational models typically employ synergy scores calculated via the Loewe method for model development^28^. The On-Screen dataset is derived from the O’Neil dataset and encompasses 4176 drug combination samples across 21 drugs and 29 cancer cell lines^25^. The synergistic scores of drug combinations are calculated using the ZIP^29^. Furthermore, the U.S. National Cancer Institute (NCI) unveiled a large dataset of anti-cancer drug combinations named NCI-ALMANAC with the aim of further expanding the number of drug combinations^22^. The NCI-ALMANAC details the synergistic effects of 104 FDA-approved drugs in pairwise combinations across 60 cancer cell lines from the NCI-60 panel. The NCI-ALMANAC dataset is available at https://wiki.nci.nih.gov/display/NCIDTPdata/NCI-ALMANAC. To ensure that only compounds with significant antiproliferative activity were included, the NCI conducted initial single-dose screenings across all 60 cell lines, followed by full dose-response evaluations for those exceeding the threshold. The NCI-ALMANAC encompasses 304,549 samples and covers synergistic 5232 drug combinations across 10 tissue types. It integrates synergy data from three distinct screening centers, each following either a 3-by-3 or 5-by-3 experimental protocol. The level of drug synergy is quantified by ComboScore, with higher values indicating stronger synergy^30^.

Unlike O’Neil and NCI-ALMANAC, which independently use drug concentrations to test drug combinations, DrugCombDB^26^ and DrugComb^27^ integrate information from multiple data sources. DrugCombDB emerged as a comprehensive repository for anti-cancer drug combinations, consolidating data from high-throughput screening, literature reviews, FDA Orange Book listings, and information on unsuccessful drug combinations^26^. In 2018, DrugCombDB encompassed 105,449 drug combination samples across 561 distinct drugs and 104 cancer cell lines. Later, the data in DrugCombDB continued to accumulate over time. By May 2019, DrugCombDB contained 448,555 combinations across 2887 distinct drugs and 124 cell lines. DrugCombDB provides synergy scores for drug combinations, which are computed based on the Bliss, HSA, Loewe, and ZIP models. The DrugCombDB dataset is available at http://drugcombdb.denglab.org.

DrugComb aggregates information from various drug synergy datasets and large-scale high-throughput screening data^27^. In 2019, DrugComb gathered 437,923 drug combinations across 93 cancer cell lines from the O’Neil, NCI-ALMANAC, FORCINA^31^, and CLOUD^32^ datasets. It should be noted that both FORCINA and CLOUD contain only one type of cancer cell line. Specifically, the FORCINA dataset includes 1181 drug combinations targeting the T98G cell line, while the CLOUD dataset comprises 40,160 drug combinations involving 283 drugs targeting the KBM-7 cell line. Through continuous crowd-sourced contributions, DrugComb has expanded dynamically over time, reaching 739,964 combinations across 8397 distinct drugs and 2320 cancer cell lines by July 2021. Comprehensive data from the O’Neil, On-Screen, NCI-ALMANAC, FORCINA, and CLOUD datasets have been fully integrated into the DrugComb repository, which is publicly accessible at https://drugcomb.fimm.fi/.

These comprehensive and integrated drug synergy datasets offer invaluable resources for developing highly reliable deep learning-based prediction models and accelerating the discovery of novel synergistic drug combinations for cancer therapy.

### Interactive data analysis portals

A number of interactive software tools and web portals have been developed to facilitate the visualization, analysis, and interpretation of drug synergy data, as summarized in Table 2.

**Table 2:** Summary of publicly interactive software tools and web portals for drug combination synergy analysis

Name	Link	Evaluation metric
Combenefit	https://sourceforge.net/projects/combenefit/	HSA, Bliss Loewe
SynergyFinder	https://synergyfinder.fimm.fi	HSA, Bliss Loewe, ZIP
DrugComb	https://drugcomb.fimm.fi	HSA, Bliss Loewe, ZIP
DeepSynergy	http://www.bioinf.jku.at/software/DeepSynergy/	Loewe
SynToxProfiler	https://syntoxprofiler.fimm.fi	HSA, Bliss Loewe, ZIP

Combenefit^33^ represents one of the earliest open-source and freely accessible platforms for the quantitative evaluation of drug combinations. It implements three classical reference models (HSA, Bliss, and Loewe) to measure drug synergy and provides an intuitive graphical interface for single and high-throughput analysis.

SynergyFinder^34–36^ is recognized as the first public web application dedicated to assessing synergistic and antagonistic drug interactions. In addition to the three models used by Combenefit, it incorporates the ZIP to provide a more comprehensive and unbiased synergy assessment. SynergyFinder offers both 2D and 3D visualization of synergy landscapes over dose-response matrices, enhancing interpretability and usability for non-expert users.

DrugComb^27^ not only provides comprehensive drug synergy data but also offers an integrated computational platform for large-scale drug combination analysis. The platform enables users to visualize and evaluate standardized dose-response data, upload and analyze their own experimental results, and assess the sensitivity of drug combinations.

DeepSynergy^37^ is a web-based predictive platform built upon a deep learning model trained with the O’Neil dataset^24^. It enables users to estimate the potential synergy of untested drug combinations within specific cell lines, using the Loewe model to quantify synergistic effects and offering a cost-efficient computational alternative to laboratory screening.

SynToxProfiler^38^ is the first online tool designed to jointly evaluate drug synergy, efficacy, and toxicity. For synergy scoring, it supports multiple reference models, including HSA, Bliss, Loewe, and ZIP, allowing flexible assessment of drug combinations. The platform prioritizes drug pairs that achieve higher efficacy with lower toxicity by integrating these metrics and is capable of handling combinations involving more than two drugs.

In summary, these interactive analysis portals provide powerful computational support for drug combination research. By enabling data visualization, synergy quantification, and predictive modeling, they significantly enhance the accessibility and reproducibility of drug synergy studies and facilitate the development of more effective combination therapies.

## Features of drugs and cell lines

**Fig. 3:**
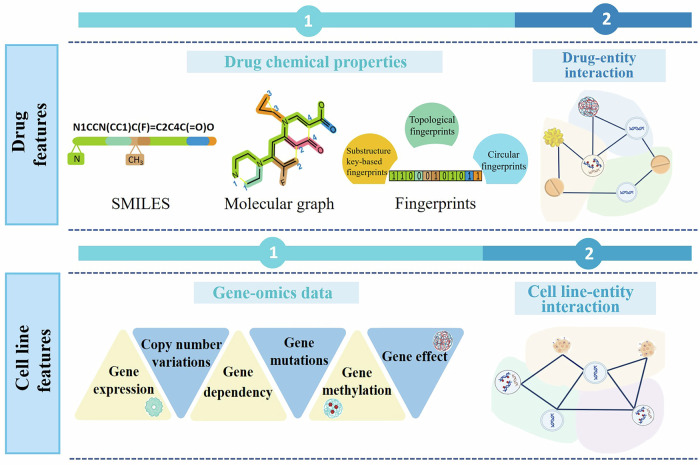
A diagram illustrating common drug and cell line features. Two common drug feature representations include: chemical information-based feature representation and drug-entity (drug/protein/cell line/enzyme/transporter) interaction-based feature representation. Two common cell line representations include: genomic data-based feature representation and cell line-entity (drug/cell line/protein/tissue) interaction-based feature representation.

For deep learning-based drug synergy prediction model, the inputs typically consist of features from both cancer cell lines and drugs. The quality of these features is crucial for the performance of prediction models. As illustrated in Fig. 3, various types of feature representations have been developed to characterize drugs and cell lines from different perspectives.

### Feature representations of drugs

For drugs, their chemical structures form the foundation of their physical and chemical properties. Drugs with similar chemical structures often exhibit similar biological activities and pharmacokinetic characteristics^39^. The chemical information of a drug can be represented as a character string, most commonly using the Simplified Molecular Input Line Entry System (SMILES) notation^40,41^. SMILES can be considered as a way to encode molecular structures as strings, which describes atoms and their connectivity in a compact text representation^42^. In computational studies, SMILES strings are often converted into molecular graphs, where atoms are treated as nodes and chemical bonds as edges^43,44^. Molecular graphs effectively capture the topological relationships within a molecule. Based on these graphs, topological fingerprints can be computed to characterize the connectivity patterns between atom pairs or atomic paths^43^. In addition, commonly used molecular fingerprints include substructure key-based fingerprints (e.g., Molecular ACCess System fingerprints^45^), which encode predefined structural fragments or functional groups, and circular fingerprints (e.g., Morgan fingerprints^46^, Extended-connectivity fingerprints^47^), which iteratively aggregate neighborhood information around each atom within a defined radius. Traditional machine learning models typically rely on such pre-computed fingerprints as input features, whereas deep learning-based methods can also learn molecular representations directly from SMILES strings or molecular graphs.

Moreover, increasing evidence suggests that the synergistic effects of drug combinations depend on interactions between drugs and various biological entities such as proteins, cell lines, enzymes, and transporters, which determine their mutual influences^48–51^. While molecular graphs capture the intra-molecular topology of a single compound, heterogeneous graphs extend this concept to inter-molecular and cross-entity relationships, linking drugs with biological entities such as proteins, cell lines, and pathways. Therefore, integrating drug-biological entity interaction information through heterogeneous graph modeling has become an important strategy for constructing comprehensive drug representations in prediction models. This approach enables the model to capture complex pharmacological associations across chemical, molecular, and cellular levels. Such multi-level representations are essential for understanding the mechanisms underlying drug synergy and for improving the biological interpretability of prediction models.

### Feature representations of cell lines

For cell lines, deep learning-based models typically employ genomic and functional data as input features, including gene expression profiles, mutation status, copy number variations, DNA methylation, gene effect scores, and dependency probabilities^52–54^. These features comprehensively characterize the molecular state and regulatory programs of each cell line, providing critical insights into its potential drug response. Such representations enable models to capture cell-type-specific characteristics and heterogeneity across cancer lineages, which are essential for predicting diverse pharmacological responses and identifying cell-line-dependent synergistic effects.

Beyond intrinsic genomic features, interactions between cell lines and biological entities such as drugs, proteins, and tissues capture multi-level regulatory mechanisms underlying disease phenotypes. For example, cell line-protein interactions can indicate pathway perturbations, while cell line-drug interactions reflect sensitivity or resistance mechanisms^55^. These interactions provide a broader biological context that links molecular alterations within cell lines to phenotypic outcomes and drug action mechanisms. By integrating both the molecular characteristics of cell lines and their interaction-based relationships within a graph framework, the model can capture molecular properties as well as system-level dependencies. This integration enhances the interpretability and performance of deep learning models in drug synergy prediction.

## Deep learning-based drug synergy prediction models

Currently, deep learning models have been extensively applied to predict synergistic drug combinations^19,56^. Table 3 provides an overview of the utilized datasets and the corresponding drug and cell line feature information employed by representative deep learning models. Specifically, existing deep learning-based prediction models can be broadly categorized into single-task learning and multi-task learning frameworks^57^. Single-task learning models focus solely on drug synergy prediction, which can be based on either branch structures or graph structures. Multi-task learning models jointly predict drug synergy along with auxiliary tasks such as drug sensitivity prediction or DDI prediction. In addition, drug synergy prediction can be formulated as either a classification task or a regression task^28,37^. In the classification setting, models categorize drug combinations as synergistic or non-synergistic according to predefined thresholds of experimental synergy metrics. The model is designed to use binary labels to distinguish between the two classes. In the regression setting, models learn to predict continuous synergy scores that indicate the strength of drug interactions across different cell lines. Table 4 provides a representative summary of deep learning models, including whether each model is single-task or multi-task, model structure design, and task formulation.

**Table 3:** Summary of datasets and feature information used by deep learning prediction models

Method	Datasets	Feature of drug	Feature of cell line
DeepSynergy^37^	O’Neil	Circular fingerprints, Physicochemical properties, Toxicophore features	Gene expression
AuDNNsynergy^63^	O’Neil	Circular fingerprints, Physicochemical properties, Toxicophore features	Gene expression, Copy number variations, Gene mutations
TranSynergy^64^	O’Neil	SMILES, Drug-protein interaction	Gene expression or gene dependency
DeepDDS^60^	O’Neil	SMILES	Gene expression
PRODeepSyn^65^	O’Neil	Circular fingerprints, Drug-protein interaction	Gene expression, Gene mutation
DTSyn^71^	O’Neil	Molecular graph, Drug-protein/drug/cell line interaction	Gene expression, Cell line-drug interaction
CGMS^57^	O’Neil	Circular fingerprints, Drug-drug/cell line interaction	Gene expression, Cell line-drug/cell line interaction
SNRMPACDC^68^	O’Neil	Circular fingerprints, Physicochemical properties, Toxicophore features	Gene expression, Copy number variations, Gene mutations
MTLSynergy^83^	O’Neil	Circular fingerprints, Molecular descriptors	Gene expression
MFSynDCP^69^	O’Neil	SMILES	Gene expression
AttenSyn^72^	O’Neil	SMILES	Gene expression
MultiComb^84^	O’Neil	SMILES	Gene expression
HypergraphSynergy^28^	O’Neil, NCI-ALMANAC	SMILES, Drug-cell line interaction	Gene expression, Cell line-drug interaction
MGAE-DC^67^	O’Neil, NCI-ALMANAC	Topological fingerprints, Circular fingerprints, Drug-drug interaction	Gene expression
HypertranSynergy^77^	O’Neil, NCI-ALMANAC	SMILES	Gene expression
MHCLSyn^78^	O’Neil, NCI-ALMANAC	SMILES	Gene expression
JointSyn^58^	O’Neil, NCI-ALMANAC	Molecular graph, Circular fingerprints	Gene expression, Somatic mutations
Kim et al.^81^	DrugComb	SMILES, Substructure key-based fingerprints	Gene expression
MatchMaker^70^	DrugComb	Chemical descriptor	Gene expression
KGE-DC^75^	DrugComb	Circular fingerprints, Drug-protein/drug/enzyme/transporter interaction	Gene expression
GAECDS^66^	DrugComb	Circular fingerprints, Drug-drug interaction	Gene annotation
MARSY^82^	DrugComb	MCF7 and PC3 signatures	Gene expression
Muthene^90^	DrugComb	Circular fingerprints, Drug-protein interaction	Gene expression
DEML^87^	DrugComb	Chemical descriptor	Gene expression
DGSSynADR^74^	DrugComb	SMILES, Drug-protein/drug interaction	Gene mutations, Gene expression
SynergyX^73^	DrugComb	Substructure key-based fingerprints	Gene expression, Gene mutations, Gene copy number, Gene methylation, Gene effect, Gene dependency
GraphSynergy^25^	On-Screen, DrugCombDB	Drug-protein interaction	Cell line-protein interaction
DeepTraSynergy^29^	On-Screen, DrugCombDB	Drug-protein interaction	Cell line-protein interaction
KGANSynergy^76^	On-Screen, DrugCombDB	Drug-protein interaction	Cell line-protein/tissue interaction
MDNNSyn^79^	On-Screen, DrugCombDB	Textual information, Drug-protein interaction, Chemical formula	Textual information, Cell line-protein interaction, Gene expression
DFFNDDS^61^	DrugComb, DrugCombDB	SMILES, Topological fingerprints	Gene expression

**Table 4:** Overview of deep learning prediction models with corresponding task settings

Method	Category	Structure	Task formulation
DeepSynergy^37^	Single-task	Branch structure	Classification, Regression
DeepDDS^60^	Single-task	Branch structure	Classification
DFFNDDS^61^	Single-task	Branch structure	Classification
AuDNNsynergy^63^	Single-task	Branch structure	Classification, Regression
TranSynergy^64^	Single-task	Branch structure	Classification, Regression
PRODeepSyn^65^	Single-task	Branch structure	Classification, Regression
GAECDS^66^	Single-task	Branch structure	Classification
MGAE-DC^67^	Single-task	Branch structure	Classification, Regression
SNRMPACDC^68^	Single-task	Branch structure	Classification, Regression
JointSyn^58^	Single-task	Branch structure	Classification, Regression
MFSynDCP^69^	Single-task	Branch structure	Classification
MatchMaker^70^	Single-task	Branch structure	Classification, Regression
DTSyn^71^	Single-task	Branch structure	Classification
AttenSyn^72^	Single-task	Branch structure	Classification
SynergyX^73^	Single-task	Branch structure	Regression
MDNNSyn^79^	Single-task	Branch structure	Classification, Regression
GraphSynergy^25^	Single-task	Graph structure	Classification
DGSSynADR^74^	Single-task	Graph structure	Regression
KGE-DC^75^	Single-task	Graph structure	Classification, Regression
KGANSynergy^76^	Single-task	Graph structure	Classification
HypergraphSynergy^28^	Single-task	Graph structure	Classification, Regression
HypertranSynergy^77^	Single-task	Graph structure	Classification, Regression
MHCLSyn^78^	Single-task	Graph structure	Classification
Kim et al.^81^	Multi-task	Drug sensitivity prediction auxiliary	Classification, Regression
MARSY^82^	Multi-task	Drug sensitivity prediction auxiliary	Classification, Regression
MTLSynergy^83^	Multi-task	Drug sensitivity prediction auxiliary	Classification, Regression
CGMS^57^	Multi-task	Drug sensitivity prediction auxiliary	Regression
MultiComb^84^	Multi-task	Drug sensitivity prediction auxiliary	Regression
DEML^87^	Multi-task	DDI prediction auxiliary	Classification, Regression
DeepTraSynergy^29^	Multi-task	DDI prediction auxiliary	Classification
Muthene^90^	Multi-task	DDI prediction auxiliary	Regression

### Single-task learning prediction models

**Fig. 4:**
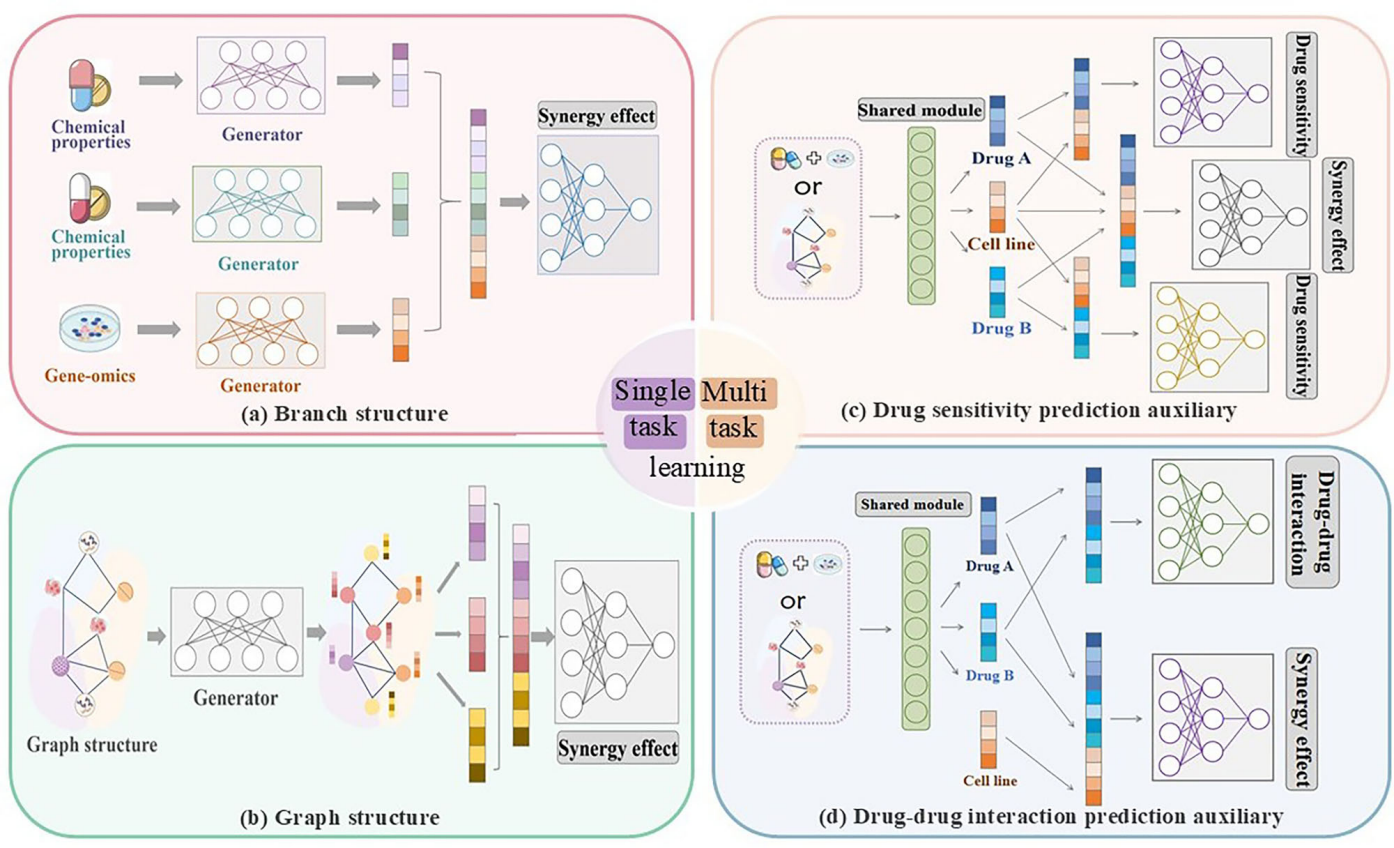
Overview of single-task learning models and multi-task learning models for drug synergy prediction. **a** Branch structure single-task learning model that independently extracts drug and cell line features. **b** Graph structure single-task learning model that learns structure-aware features from entity relationship graphs. **c** Multi-task learning model integrating drug sensitivity prediction as an auxiliary task. **d** Multi-task learning model incorporating drug-drug interaction prediction as an auxiliary task.

Single-task learning prediction models can be categorized into branch structures or graph structures according to their architectures. Branch structures enable these models to process multiple features or information sources in parallel, which effectively captures the relationships among various inputs related to drugs and diseases. In contrast, graph structures are commonly employed to manage complex relationships within heterogeneous networks that reveal the interactions between drugs and diseases. These two types of single-task learning model architectures are illustrated in Fig. 4a, b, respectively.

#### Branch structure-based prediction models

Branch structures are currently the architecture most commonly used in drug synergy prediction models^20,58^. These structures allow models to independently learn the features of drugs and cell lines from different branches, which is crucial for understanding how drugs interact with each other and how they collectively affect cell lines^56^. Branch structure-based models can be categorized into three types based on the feature extraction method^59^: models which independently learn the features of drugs and cell lines, models which construct drug combination features based on relationships between drug pairs, and models which learn the features of drugs and cell lines by capturing interactions between them.

DeepSynergy^37^ is a typical prediction model with a branch structure that independently learns the features of drugs and cell lines. DeepSynergy directly utilizes chemical descriptor information of drugs and gene expression data of cell lines to construct drug and cell line features, respectively. To construct better drug features, DeepDDS^60^ employs graph neural networks to learn informative drug features based on drug molecular graphs, which help to capture the complex patterns of drug molecular structures. Besides, DFFNDDS^61^ uses a BERT model^62^ to convert drug SMILES into vectors, which combine with drug hashed atom pair fingerprints to form the drug features. Both DeepDDS and DFFNDDS utilize multi-layer perceptron to learn cell line features from gene expression data. For generating better cell line features, AuDNNsynergy^63^ utilizes three autoencoders to learn cell line features from gene expression, copy number variations, and gene mutation data. TranSynergy^64^ uses gene expression and gene dependency profiles to construct the features of cell lines, and the target gene of drugs is utilized by TranSynergy to construct the drug features. PRODeepSyn^65^ extracts the hidden state matrix of genes from the PPI network, which combines with cell line gene expression embeddings to generate cell line features. This method of constructing cell line features can approximate the explicit state vectors of genes and comprehensively capture the biological characteristics of the cell lines. The aforementioned models focus primarily on the ability to independently learn the features of individual drugs and cell lines. Meanwhile, some branch structure-based models have begun to use the interaction information between drugs to construct features for drug combinations.

As a representative branch structure model using interactions between drugs, GAECDS^66^ utilizes a graph autoencoder to learn drug features from the synergy interactions between drugs. However, GAECDS solely utilizes the prior synergistic relationships between drugs to learn drug features, which may limit the model in capturing the complexity of drug interactions. For fully reflecting relationships among drugs, MGAE-DC^67^ represents the interactions between drugs in a cell line as a synergistic graph, an additive graph, and an antagonistic graph, where the synergistic (additive/antagonistic) graph includes the synergistic (additive/antagonistic) interactions between drugs. A multi-channel graph autoencoder is employed to extract features of drugs from graphs specific to a cell line, and an attention mechanism is then integrated to synthesize cell-line specific features for each drug. In fact, the drug combination features in GAECDS^66^ and MGAE-DC^67^ are still directly concatenated from the individual drug features learned based on drug interaction relationships. Besides, the relationships among drugs can also be explored from the aspect of drug properties. SNRMPACDC^68^ utilizes siamese convolutional networks and random matrix projection technology to process the chemical information of two drugs, which are converted into a drug combination feature via the Hadamard product. Compared to drug combination features formed by concatenating individual drug features, the drug combination features constructed by SNRMPACDC enhance its prediction performance. To generate more reliable drug combination features, JointSyn^58^ connects the molecular graphs of two drugs into a joint graph and learns a feature of drug combination through graph attention network. MFSynDCP^69^ designs a graph aggregation module with an adaptive attention mechanism, which can dynamically focus on key information within molecular graphs of drug pairs and comprehensively capture important interaction information between molecular graphs to generate features. Although such branch structured models use relationships between drug pairs to generate drug combination features, they still construct drug features and cell line features separately.

The models in the third class focus on learning the features of drugs and cell lines by analyzing hidden interactions between drug chemical information and genomic information. MatchMaker^70^ concatenates the chemical descriptor of a drug and gene expression profile of a cell line, and further employs a fully connected networks (FCNs) to extract the drug feature conditioned on gene expressions. In addition, DTSyn^71^ uses a multi-grained transformer encoder to fully extract the features of both drugs and cell lines. The fine-grained transformer encoder is designed to capture the associations between drug chemical substructures and genes, as well as interactions among genes. Meanwhile, the coarse-grained transformer encoder focuses on extracting the interactions of drug chemical structures-drug chemical structures and drug chemical structures-gene expressions. AttenSyn^72^ incorporates gene expression features of cell lines into the feature matrix of drug molecular graphs to learn multi-resolution features of drugs. A focus-based pooling module is then employed to learn the interaction information between drug pairs, which aids in enhancing the expressive power of drug combination features. Both drug-cell line interactions and DDIs are utilized to construct drug features. The aforementioned models can only learn the static interactions between drugs and cell lines; SynergyX^73^ designs attention modules to dynamically capture cross-modal interactions between drugs and cell lines. SynergyX constructs mutual attention and self-attention modules that use original representations of drugs and cell lines to calculate the attention from cell lines to drugs and from drugs to cell lines, which is used to dynamically update the features of drugs and cell lines.

#### Graph structure-based prediction models

In recent years, graph structure-based prediction models mainly extract the features of drugs and cell lines based on specifically designed graphs, such as heterogeneous graphs and hypergraphs^59^. GraphSynergy^25^ predicts synergistic drug combinations on a PPI network, where each drug or cell line aggregates the information of its related *H*-hop proteins in the PPI network by inner product operations. Besides, DGSSynADR^74^ constructs a drug-protein relationship graph, and employs a low-rank global attention mechanism to weight and aggregate node information for globally learning features of drug nodes. To enrich the features of drugs and cell lines, KGE-DC^75^ builds a heterogeneous graph to reflect drug-drug synergy relationships, and drug-targets/enzymes/transporters interactions. In addition, KGANSynergy^76^ constructs a heterogeneous graph that includes interaction information of drug-protein, cell line-protein, cell line-tissue, and protein-protein.

In a hypergraph, a hyperedge connects multiple nodes, which can accurately reflect the multi-way relations between drug combinations and cell lines. HypergraphSynergy^28^ formulates synergistic drug-drug-cell line triplets as a drug synergy hypergraph to represent multi-way relations, and learns the features of drugs and cell lines by employing a hypergraph neural network. To fully utilize the information in drug synergy hypergraphs, HypertranSynergy^77^ extracts coarse-grained information from the drug synergy hypergraph using a transformer-based approach in the coarse-grained information extraction module and constructs a similarity matrix in the fine-grained information extraction module to supplement fine-grained information. MHCLSyn^78^ employs a multi-view hypergraph contrastive learning approach on the drug synergy hypergraph and hypergraphs resulting from edge perturbation processing on drug synergy hypergraph, which allows for more expressive and discriminative node representation learning.

In addition to the aforementioned two types of prediction models, the multi-modal deep learning model MDNNSyn^79^ is also designed to predict synergistic drug combinations. The model primarily adopts a branch structure to learn multi-modal features, within which a hypergraph structure is used to extract topological modal features of drugs and cell lines.

### Multi-task learning prediction models

Single-task learning models focus solely on predicting synergistic drug combinations, while multi-task learning models apply information from auxiliary tasks to enhance the performance of drug synergy prediction. Drug sensitivity prediction and DDI prediction are often used as auxiliary tasks in drug synergy prediction models. The architectures of these two types of multi-task learning models are illustrated in Fig. 4c, d, respectively.

#### Drug sensitivity prediction auxiliary

Drug sensitivity information can describe the sensitivity of cells to specific drugs^80^, which lays the foundation for assessing the synergistic effects of drug combinations. Both Kim et al.^81^ and MARSY^82^ utilize FCNs to construct multi-task deep neural networks for predicting drug sensitivity and drug synergy, simultaneously. Kim et al.^81^ utilize multi-task deep neural networks to process molecular, genetic, and phenotypic features of drugs and cell lines for predicting single drug sensitivity and drug synergy on cell lines. MARSY^82^ uses gene expression profiles of cell lines and differential expression features induced by each drug as input, and employs two FCNs to learn the representations of drug-drug-cell line triplets and drug pairs. Both representations are concatenated and used as inputs to a multi-task predictor, which can generate the drug synergy and single drug sensitivity scores. However, multi-task deep neural networks are primarily built around FCNs, which limits the ability of model to learn shared features across different tasks. MTLSynergy^83^ and CGMS^57^ utilize autoencoders and heterogeneous graph attention networks to optimize shared features by leveraging the relationships between two tasks. Unlike the aforementioned models that use single drug sensitivity prediction as an auxiliary task, MultiComb^84^ utilizes sensitivity prediction of drug combinations as an auxiliary task. MultiComb employs the attention mechanism to construct shared features for two distinct tasks, and uses the cross-stitch mechanism to learn relationships between tasks. Drug combination sensitivity and synergy scores serve as critical metrics for identifying the optimal drug combination tailored to a specific cancer cell line.

#### Drug-drug interaction prediction auxiliary

Drug synergy is closely related to DDIs, and can even be considered a specific type of DDIs^85,86^. Drug synergy prediction and DDI prediction both utilize features of two drugs, so DDI prediction serves as an auxiliary task that can help the drug synergy prediction model generate more informative drug features. DEML^87^ designs a hybrid ensemble layer structure to construct higher order drug features for drug synergy and DDI prediction. DEML also has a task-specific fusion layer that merges features for each task using a gating mechanism. As a specific type of DDIs, drug-drug toxicity and side effects can precisely indicate the adverse outcomes of drug combinations. Predicting drug-drug toxicity or side effects as an auxiliary task helps the drug synergy prediction model to identify safe and reliable synergistic drug combinations^88,89^. DeepTraSynergy^29^ designs a transformer to process drug-target and protein-protein interaction information, which can generate shared drug features for drug synergy and drug-drug toxicity prediction. Notably, the transformer provides context-aware representations for every position in the drug molecule, focusing on segments with the highest predictive efficiency. Muthene^90^ applies meta-paths to represent the presumed interaction paths between the drug pairs and their shared targets. Drug embeddings learned from meta-paths, along with chemical property features, form the shared drug features used for drug synergy and drug-drug adverse effect prediction.

Compared to most single-task learning models, multi-task learning models have a relatively simple architecture and fewer modules. The addition of auxiliary tasks enhances performances of multi-task learning models. Ablation studies on multi-task learning models further confirm the significance of auxiliary tasks.

### Comparison between different types of models

To compare the prediction performance of different types of models, we select several single-task learning models with branch structures (DFFNDDS^61^, MGAE-DC^67^, SynergyX^73^) and graph architectures (DGSSynADR^74^, HypertranSynergy^77^), multi-modal deep learning model (MDNNSyn^79^), as well as multi-task learning models (MARSY^82^, Muthene^90^) incorporating drug sensitivity prediction auxiliary and DDI prediction auxiliary. The performance of these models is relatively outstanding among their peers. Due to the fact that these prediction models utilize different datasets, the scale, quality, and characteristics of datasets can all impact the performance of models. Therefore, it is difficult to directly compare the performance of models. To overcome this difficulty, we conduct unbiased experiments using the same data for comparable models. Currently, DrugComb dataset is already the largest public dataset of drug combinations and encompasses all the information from other datasets. Hence, we use the DrugComb dataset as data source to conduct comparative experiment. Drug synergy prediction can be considered a classification task or a regression task^91^, so the performance of models on both tasks needs to be compared.

**Fig. 5:**
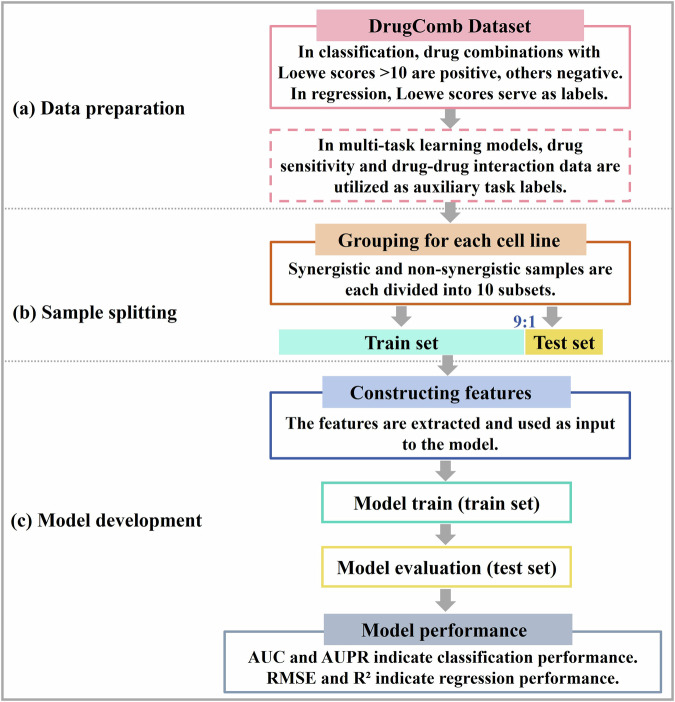
The complete workflow followed by all models for drug synergy prediction comparison encompasses three main stages. **a** Data preparation: the DrugComb dataset is utilized, with Loewe scores determining classification labels (scores > 10 as positive samples, others as negative samples) or serving as regression targets. In multi-task learning models, drug sensitivity and drug-drug interaction data are utilized as auxiliary task labels. **b** Sample splitting: synergistic and non-synergistic samples for each cell line are separately partitioned into 10 subsets, with a 9:1 ratio applied to construct the training and testing sets. **c** Model development: drug and cell line features are extracted, models are trained using the training set, and their performance is evaluated on the test set. AUC and AUPR are used to assess classification performance, while RMSE and *R*^2^ are used for regression.

We set the threshold of 10 for the Loewe score when performing classification because it is widely recognized in the drug synergy research community that scores above 10 reliably indicate synergistic drug combinations. Scores between -10 and 10 are considered additive, and scores below -10 are considered antagonistic. According to the SynergyFinder user guide^34–36^, the summary synergy score represents the average excess response due to drug interactions. Choosing 10 as the positive sample threshold reduces the influence of experimental noise, ensures a clear distinction between positive and negative samples, and balances sample numbers for reliable model training. Therefore, drug combinations with Loewe scores above 10 are labeled as positive samples, and the rest as negative samples. Hyperparameters used in these models are determined by 10-fold cross validation (10-CV). By ensuring that the distribution of categories is uniform in each fold during the 10-CV process, it can reduce the bias in the model evaluation process and make the model evaluation results more reliable. Specifically, stratified sampling is performed within each cell line, where synergistic and non-synergistic drug combinations are independently divided into 10 equal-sized subsets. This ensures that each subset preserves the balanced distribution of positive and negative samples. For each iteration of the 10-CV, one subset from both categories across all cell lines is aggregated to form the test set, while the remaining samples constitute the training set. We use two evaluation metrics to compare the classification performance: area under the receiver operating characteristic curve (AUC) and area under the precision-recall curve (AUPR). Additionally, the two metrics root mean square error (RMSE) and coefficient of determination (*R*^2^) are used to compare the performance of models in regression task. The performance of the models is represented by the average values obtained from ten executions of 10-CV. To provide a clear overview of the model comparison, Fig. 5 illustrates the complete workflow followed by all models, including data preparation, sample splitting, and model development.

As shown in Table 5, the performance of MARSY and Muthene is superior to that of DFFNDDS, MGAE-DC and DGSSynADR, comparable to HypertranSynergy, and inferior to SynergyX and MDNNSyn. SynergyX and MDNNSyn stand out from other models due to their ability to construct informative features for both drugs and cell lines. SynergyX converts drug SMILES into substructure-partitioned fingerprint spectra to extract substructural features of drugs. Compared to the drug features used by other models, substructural features can more finely capture local chemical patterns and functional groups in drug molecules, which provides more precise molecular-level information. SynergyX applies gene expression, gene mutations, gene copy number, gene methylation, gene effect and gene dependency probability to construct cell line features. SynergyX dynamically captures the interactions between drugs and cell lines to update the features of drugs and cell lines. MDNNSyn utilizes textual information, target data, and chemical formulas from drugs, as well as textual information, target data, and gene expression profiles from cell lines to construct the initial representations for them. Besides, MDNNSyn learns the multi-modal features of drugs and cell lines from textual information, target data, and chemical formulas of drugs, as well as textual information, target data, and gene expression profiles of cell lines. Different modality features of drugs and cell lines are respectively synthesized into their final features through a gating mechanism.

**Table 5:** Performance comparison of prediction models on DrugComb dataset

Model	Category	AUC	AUPR	RMSE	*R*^2^
DFFNDDS	Single-task	0.832	0.566	15.11	0.572
MGAE-DC	Single-task	0.845	0.624	13.35	0.648
SynergyX	Single-task	0.907	0.683	12.94	0.686
DGSSynADR	Single-task	0.841	0.583	14.66	0.598
HypertranSynergy	Single-task	0.866	0.647	13.21	0.654
MDNNSyn	Single-task	0.902	0.686	13.08	0.676
MARSY	Multi-task	0.871	0.654	13.75	0.613
MARSY w/o aux	Single-task	0.813	0.563	15.17	0.560
Muthene	Multi-task	0.877	0.662	13.15	0.669
Muthene w/o aux	Single-task	0.842	0.627	13.46	0.642

Note: “aux” stands for “auxiliary task”.

In comparison with single-task learning models, MARSY and Muthene use less data information to construct features for drugs and cell lines. However, the prediction performance of them is still not inferior to other single-task learning models except for SynergyX and MDNNSyn. We use MARSY w/o aux and Muthene w/o aux to denote the models MARSY and Muthene after removing their auxiliary tasks. As presented in Table 5, the performance of models MARSY and Muthene deteriorates when their auxiliary tasks are removed. Hence, the addition of auxiliary tasks is crucial for enhancing prediction performance of MARSY and Muthene. The key to SynergyX and MDNNSyn performing well lies in their ability to construct high-quality features. The quality of features, especially the quality of shared features, is also crucial for multi-task learning models. Therefore, multi-task learning models should further enhance the ability to construct shared drug or cell line features with richer information.

## Discussion and outlook

**Fig. 6:**
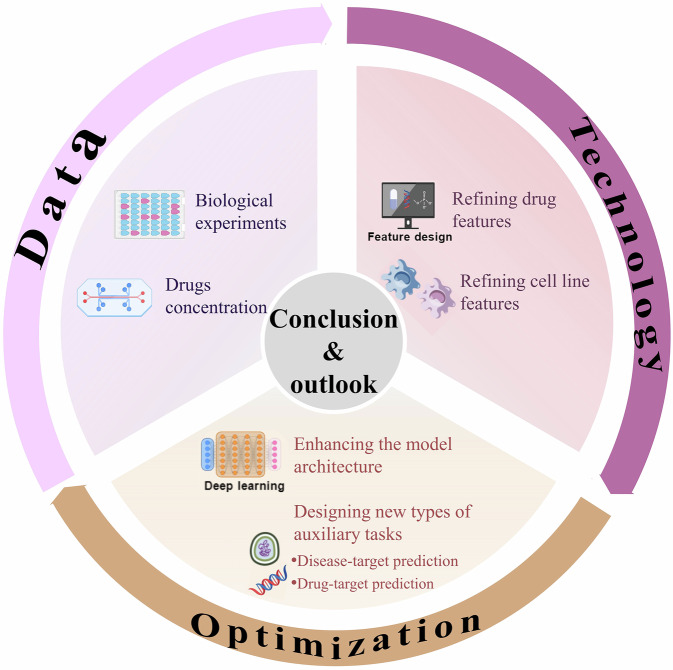
The outlook of deep learning-based drug synergy prediction models. The prediction models can be improved from three aspects: data, technology, and optimization.

Drug synergy therapy is considered as a promising approach to improve the effectiveness of cancer treatment, which is generally capable of overcoming drug resistance and side effects. However, identifying all possible synergistic drug combinations is infeasible due to the huge size of drug combinatorial space. Practically, a deeper understanding of the underlying biological impact is required to efficiently explore the extensive synergistic drug combinations. Deep learning-based models have increasingly become a pivotal component in drug synergy prediction research, which help to integrate data from diverse biomedical sources and clarify their impacts on the synergistic drug combinations discovery. In this context, we discuss the latest deep learning models for drug synergy prediction. We also pay special attention to the fact that existing deep learning-based prediction models can be divided into single-task learning models and multi-task learning models. Single-task learning models focus on predicting drug synergy tasks. Multi-task learning prediction models can leverage the correlations between drug synergy prediction task and auxiliary tasks by sharing parameters, which enables the model to predict different tasks. Current studies and benchmark datasets mainly focus on cancer. However, these deep learning-based approaches can also be applied to other disease indications, such as cardiovascular or infectious diseases, when relevant data exist. Deep learning-based prediction models have demonstrated tremendous potential in predicting the synergistic effects of drug combinations, but they still have room for improvement in data, technology, and optimization, as illustrated in Fig. 6.

### Data: measuring the concentration of drugs

Currently, deep learning-based models in drug combination therapy primarily determine whether two drugs have a synergistic effect. These models usually only screen for potentially effective drug combinations based on the types of drugs. However, they do not directly consider the concentration of drugs, which is an important factor in practical applications. During the laboratory verification phase, researchers need to measure and adjust the concentration of the drug reagents through biological experiments to ensure that the drug combinations work best at the appropriate concentrations. This process is not only time-consuming but also requires considerable experimental resources and expertise^92^. To enhance the practicality and accuracy of the models, future development directions could include incorporating drug concentration as a key parameter. This means the models would need to predict not only which drug combinations have a synergistic effect but also the effects of these drugs at different concentrations and how to adjust the concentrations for the best therapeutic effect. This will involve constructing a drug synergy dataset that includes drug concentration information, so that the prediction models can process and learn the relationship between drug concentration and therapeutic efficacy.

### Technology: refining drug and cell line features

Most deep learning approaches often analyze drug and cell line features in isolation. However, genetic differences between cell lines, such as gene mutations and copy number variations, can lead to variations in drug sensitivity^93^. Additionally, the expression levels of drug targets may vary across cell lines, directly affecting drug efficacy^94^. Furthermore, the interactions between different drugs can have varying effects in different cell lines, which will influence the overall treatment outcomes. Hence, capturing relationships between drugs and cell lines to refine drug and cell line features can yield more insightful information for prediction models. Future research directions may include developing novel algorithms to better understand and model the complex interactions between drug combinations and cell lines, which can provide guidance for constructing more reasonable drug and cell line features. Within the multi-task learning framework, shared features can help the model simultaneously perform the drug synergy prediction task and auxiliary tasks. In addition, different tasks can share the common features obtained during the representation learning process, while also learning task-specific features. This complementarity helps enhance the understanding of models on drug synergies. Therefore, designing new technology to enhance the quality of shared features in multi-task learning prediction models is also a direction worth exploring.

### Optimization: improving multi-task prediction

Currently, an increasing number of deep learning-based prediction models are employing multi-task learning to enhance their performance in drug synergy prediction. These approaches, which combine multi-task learning with deep learning, can handle multiple related tasks simultaneously and improve the generalization capability and predictive accuracy of models through shared drug and cell line representations. Specifically, prevalent auxiliary tasks include drug sensitivity/response prediction and drug-drug interaction prediction. Additionally, predicting drug-drug toxicity, a specialized aspect of drug-drug interaction prediction, is also utilized as an auxiliary task. The prediction models enhances their performances in drug synergy prediction tasks by assimilating knowledge from these auxiliary tasks. However, multi-task learning models still have room for improvement.

#### Enhancing the model architecture

In multi-task learning prediction models, the total loss is often calculated by directly summing the losses of drug synergy prediction task and auxiliary tasks, which may not be the optimal solution for calculating the total loss in multi-task learning models. Hence, finding the optimal weight parameters for each loss function and determining how to combine multiple loss functions to achieve the best overall performance are questions worth exploring when constructing multi-task prediction models. Besides, in some multi-task learning models, such as MTLSynergy^83^, the performance of the single-drug sensitivity prediction task has not been directly compared with previous studies. The reason for this lack of direct comparison is the variation in datasets, sensitivity scoring metrics, and evaluation indicators used across different studies. Hence, future research is expected to address these differences to enable more meaningful comparisons in auxiliary tasks.

#### Designing new types of auxiliary tasks

In fact, designing new auxiliary tasks is also necessary. By introducing novel auxiliary tasks, the model can understand the synergistic effects of drug combinations from multiple perspectives and levels, and learn richer and more diversified features.

##### Disease-target association prediction auxiliary

By incorporating disease-target association prediction as an auxiliary task, models can identify new potential therapeutic targets that may not have been previously considered. This task helps bridge the gap between understanding disease biology and developing drug synergy therapeutic strategies^95,96^. Besides, disease-target association prediction can uncover interactions between drugs and novel disease targets, which can guide the combination of drugs^97^. Furthermore, when multiple targets belong to the same disease pathway or interconnected pathways, drug combinations targeting these targets may exhibit synergistic effects.

##### Drug-target affinity prediction auxiliary

Drug-target affinity (DTA) prediction offers another promising auxiliary task by identifying targets with high affinity for known drugs. DTA prediction as an auxiliary task can uncover potential effective targets for drug combinations and enhance the precision of drug synergy prediction^98^. Predicting binding affinities can help models identify synergistic drug combinations by ensuring that selected targets are biologically relevant and by ranking potential interactions based on their likelihood of contributing to synergistic effects. In addition, DTA prediction can reveal subtle differences in binding profiles among structurally similar drugs, which aids the models in predicting rational synergistic drug combinations^99^.

In summary, designing new auxiliary tasks helps multi-task learning models to gain a deeper understanding of drug synergy mechanisms from multiple perspectives and enhances the accuracy of prediction results. Therefore, future research could also focus on designing more effective and interpretable auxiliary tasks within multi-task learning models.

## Data Availability

No datasets were generated or analysed during the current study.
